# Lead Emissions and Population Vulnerability in the Detroit (Michigan, USA) Metropolitan Area, 2006–2013: A Spatial and Temporal Analysis

**DOI:** 10.3390/ijerph14121445

**Published:** 2017-11-23

**Authors:** Heather Moody, Sue C. Grady

**Affiliations:** 1 Department of Environmental Science, Siena Heights University, 1247 East Siena Heights Drive, Adrian, MI 49221, USA; 2 Department of Geography, Environment, and Spatial Sciences, Michigan State University, 673 Auditorium Road, Room 207, East Lansing, MI 48824, USA; gradys@msu.edu

**Keywords:** lead emissions, lead deposition, environmental justice, AERMOD, Detroit, Michigan, USA

## Abstract

*Objective*: The purpose of this research is to geographically model airborne lead emission concentrations and total lead deposition in the Detroit Metropolitan Area (DMA) from 2006 to 2013. Further, this study characterizes the racial and socioeconomic composition of recipient neighborhoods and estimates the potential for IQ (Intelligence Quotient) loss of children residing there. *Methods*: Lead emissions were modeled from emitting facilities in the DMA using AERMOD (American Meteorological Society/Environmental Protection Agency Regulatory Model). Multilevel modeling was used to estimate local racial residential segregation, controlling for poverty. Global Moran’s I bivariate spatial autocorrelation statistics were used to assess modeled emissions with increasing segregation. *Results*: Lead emitting facilities were primarily located in, and moving to, highly black segregated neighborhoods regardless of poverty levels—a phenomenon known as environmental injustice. The findings from this research showed three years of elevated airborne emission concentrations in these neighborhoods to equate to a predicted 1.0 to 3.0 reduction in IQ points for children living there. Across the DMA there are many areas where annual lead deposition was substantially higher than recommended for aquatic (rivers, lakes, etc.) and terrestrial (forests, dunes, etc.) ecosystems. These lead levels result in decreased reproductive and growth rates in plants and animals, and neurological deficits in vertebrates. *Conclusions*: This lead-hazard and neighborhood context assessment will inform future childhood lead exposure studies and potential health consequences in the DMA.

## 1. Introduction

In the United States and worldwide, hazardous waste sites and polluting industries are disproportionately located in areas with a high proportion of poor and minority populations [1,2]; a phenomenon referred to as “environmental injustice”. Environmental injustices appear to be consistent across a variety of stakeholders under similar structural conditions. For example, in the Northeast and Midwest regions of the United States, disinvestment in older industrial cities has resulted in depressed urban communities, typically minority communities characterized by high unemployment, low-tax base, declining public infrastructure and retail flight to suburban areas. Residents living in such communities have limited bargaining power—even with community organized resistance—to prevent polluting industries from purchasing low-cost land [2,3,4]. Black residents in particular, living in hypersegregated and poor urban communities with discriminatory zoning and land-use practices [2,4] and limited social mobility are unable to explore external economic opportunities, contributing to concentrated poverty and more hazardous industrial-related employment opportunities.

In the Detroit Metropolitan Area (DMA), studies have found a similar pattern despite great opposition. In 1998, Mohai and Bryant [5] showed that the proportion of black and/or low-income residents increased with proximity to polluting industrial facilities, commercial hazardous waste facilities, and abandoned/uncontrolled hazardous waste sites. In addition, low-income black communities in Detroit’s east and southwest sides were significantly more burdened with traffic particulate matter (PM_10_, PM_2.5_) [6] compared to other parts of the city. In 2006, Wu and Batterman [7] revealed that non-white, K-12 schools located in impoverished neighborhoods of Detroit were more likely to be exposed to auto and commercial truck traffic emissions compared to non-impoverished neighborhoods. Downey [8] also found that Detroit’s black neighborhoods were disproportionately burdened with toxic releases as reported in the Environmental Protection Agency’s (EPA) Toxic Release Inventory. Furthermore, Lee and Mohai [9] determined that EPA designated brownfield sites were most likely to be in impoverished, minority neighborhoods in the DMA. Moreover, Smith [10] showed that the DMA’s poor and black neighborhoods were strong predictors for EPA’s Superfund site locations. Lastly, Moody et al. [11,12] found that in the DMA, black segregated neighborhoods with lower socioeconomic characteristics were strong predictors of elevated blood lead levels (BLLs) in children residing in those areas.

Lead in an important toxin in old industrial urban environments—the source of which may be from historically emitted leaded gasoline, smelters, iron and steel production, lead-acid-battery manufacturing, nonferrous (brass and bronze) foundries, landfills, waste incinerators, sewage sludge incinerators, hazardous waste sites, power plants (refineries/coal burning), and older homes that contain lead based paint/pipes [13]. It is well established that air lead and deposition levels can be elevated in areas without adequate air emission control equipment from coal burning power plants [14], coking operations [15], iron and steel manufacturing [16,17], oil refining [16,18], smelting [17,19,20], and waste incineration [16,17]. Importantly, each of these industry types emits lead into the DMA’s City of Detroit located in Wayne County. There is also evidence of siting selection preference for these facilities in segregated and low-income and/or minority communities in addition to unequal enforcement of their air emissions permits [18,21,22,23].

Residents living near these industrial facilities or working in them have greater potential exposure to lead laden air and dust. Children are most susceptible because of their small size and developing physiology and may inhale lead from the air and ingest lead from water and/or soil (dermal absorption is negligible) in places where lead accumulates [13]. The re-suspension of soil/particle lead, both indoors and out, from lead laden soils is especially prevalent in inner-city neighborhoods such as Detroit [24,25] and is evidenced by a strong seasonal component of increased BLLs in children, particularly exposed in the summer months [24,26,27].

Three case studies add to the environmental justice lead literature and evidence unequal enforcement of air emissions permits in depressed minority/poor urban centers of Detroit. Beginning the fall of 2012, Governor Rick Snyder’s Michigan Economic Development Corporation (MEDC) successfully lobbied the Michigan Department of Environmental Quality (MDEQ) for relaxed air permits for one of Detroit’s leading lead emitters, Severstal Steel of Dearborn (acquired by AK Steel in September 2014). The MEDC requested that Severstal Steel be allowed to operate (grandfathered) under 2006 MDEQ permit rules ([28], Amy Banninga, MEDC, email communication, 10 September 2012) as opposed to the current and stricter state air emission standards of 2008. This special permit allowed for greater emissions of mercury, sulfur dioxide, and lead; from 0.01726 pounds (7.83 g) per hour to 0.03589 pounds (16.28 g) per hour (151.1976 lb/year to 314.3964 lb/year or 68.58 kg/year to 142.61 kg/year) of lead for all emission units (C Blast Furnace Casthouse Baghouse Stack, Desulfurization Baghouse, B&C Casthouses, B&C Stove Stacks), above even the 2006 regulations. These emission levels would have violated their existing 2008 permit ([29], LLC Permit to Install Application 182-05C). This request came after Severstal was unable to achieve compliance with their 2008 permit limits ([30], Lynn Fiedler, DEQ, email communication, 16 August 2012). In lack of support for this relaxed permit, an MDEQ inner office email from the Air Quality Division Assistant Chief detailed the following: at this time, Severstal has received 117 citizen complaints alleging fallout/opacity, 76 MDEQ on-site visits, and over twenty MDEQ and federal EPA violation notices for ongoing exceedances including those for lead (Lynn Fiedler, DEQ, email communication, 16 August 2012). Severstal, now AK Steel, is in the southern portion of Dearborn in a neighborhood of poor, mostly Arab immigrants and less than one mile from Salina Elementary and Intermediate Schools [31]. In May 2015, the Justice Department charged AK Steel with 42 air pollution violations alleged by the MDEQ. As a result, they paid 1.35 million dollars in fines and installed air filtration systems at the Salina Elementary and Intermediate schools near the plant but still operates under the same permit [32].

While AK Steel was issued less stringent air permits (May 2014), the Detroit Marathon Oil Refinery located in Southwest Detroit, also received a revised air permit allowing increased emissions, including lead, because of processing tar sand oil imported from Canada [28,33]. Marathon too has unresolved MDEQ air emissions violations including the allowance of more sulfur dioxide than the old permit allowed [28].

In 2003 a five-part series and cover story [34] run by the Detroit Free Press investigated Master Metal, a battery smelter plant owned by NL Industries. This plant emitted illegal amounts of lead dust for two decades [35] exposing eight schools, 16 parks, and 5000 children under five years of age in Detroit’s segregated poor and minority east side to lead dust [35] before ceasing operations in 1984. After minimal testing and cleanup by the EPA, the investigation was closed [35]. In response, the Detroit Free Press hired soil experts to test 97 samples up to 2.9 km from the vacated smelter site and found contamination in 10 neighborhood yard samples at levels as high as 5811 ppm [35]. These levels exceeded many EPA health regulations and would require the EPA to initiate cleanup by its own standards of 400 ppm [35,36]. Unfortunately, NL Industries declared bankruptcy and due to a lack of remediation funding, the neighborhoods remain contaminated today [35,37]. Lack of enforcement and compliance with cleanup standards in segregated low-income and/or minority communities is one hallmark of environmental injustice [21,22,23].

Scientists have found no safe level of lead in children. The most common physiological effects of lead poisoning are neurological and neurobehavioral, lower IQ and slowed growth and anemia [13,38,39,40,41,42,43,44,45,46]. The smallest detection limit achieved, 0.005 micrograms of lead per deciliters of blood (μg/dL), was established by the Centers for Disease Control and Prevention (CDC) using atomic absorption spectrometry. Children having no exposure history will yield a non-detect result [13]. Prior to January 2013, 10 μg/dL was used to determine a high level of blood concentration. In 2013, that level was changed to 5 μg/dL by the CDC. Over the last two decades substantial research [13,38,39,40,41,42,43,44,45,46] has supported that even lower levels of lead in the blood can cause permanent physiological damage to the neurological system. There is a need therefore, to model this environmental hazard associated with industrial emissions and the potential injustices in relation to location of residence.

Few studies have modeled industrial lead emissions and geospatially linked the emissions to underlying neighborhoods in an attempt to characterize resultant environmental health risk. The purpose of this research is to: (1) identify areas of elevated airborne lead and depositional emissions in the DMA from 2006 through 2013, (2) characterize the racial residential segregation of the recipient neighborhoods and (3) estimate IQ loss from airborne lead in children of recipient neighborhoods. It is hypothesized that in neighborhoods of high black segregation, airborne and depositional emissions of lead will be the highest. The findings from this research will provide the lead-hazard and neighborhood contexts assessments from which to study childhood lead exposures and potential health consequences in the DMA.

## 2. Materials and Methods

### 2.1. Study Area and Population Vulnerability

**Figure 1:**
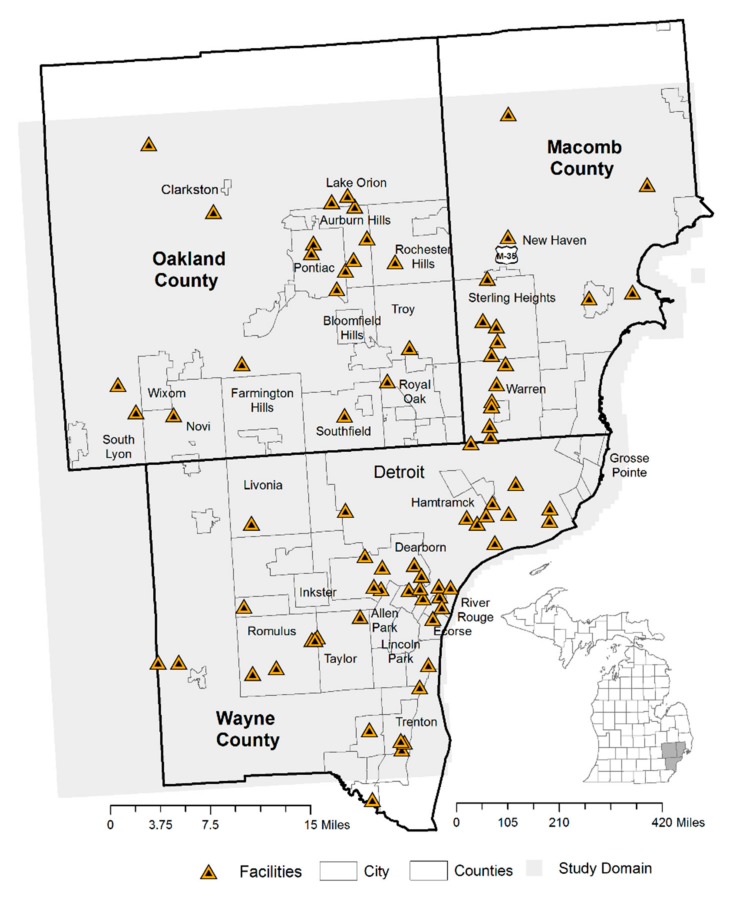
Reference map—locations of lead emitting facilities, AERMOD domain and city boundaries in the Detroit Metropolitan Area, 2006–2013.

This study was conducted in the Detroit Metropolitan Area (DMA) in southeast Michigan, USA. The DMA consists of three counties, Wayne, Macomb, and Oakland, equal to a population of 3,734,090 and an area of 2152 square kilometers [47] (see Figure 1).

The city of Detroit is the center of the three-county urban cluster and located in Wayne County with a population of 713,862 in 2010 [48]—a 25.0% decrease from 2000 following the recession and housing foreclosure crisis [49]. The cities of Dearborn (*n* = 98,626) and Pontiac (*n* = 59,284), the later located in Oakland County are the second and third largest cities in the DMA. The racial makeup of the DMA is 50% white, 38.5% black and 11.5% other. Comparatively, the racial makeup of Detroit has a high black population of 82.7% and white population 10.6% including a large white-Arab population in the southwestern suburbs of Dearborn and Dearborn Heights [48]. This ethnicity is often overlooked but important to this study as a minority and immigrant group facing environmental discrimination. In 2010, Detroit was also the poorest city in the country with 39.3% of residents living in poverty (Wayne County 24.0%, Macomb County 12.2% and Oakland County 9.9%). [47]. The high percentage of black population and low income of residents in Detroit reflects a concentration of poverty—i.e., neighborhoods of severely disadvantaged social environments with reduced life chances [50,51] and increased vulnerability to poor health.

This study of environmental injustice utilized a multilevel modeling approach to estimate racial residential segregation [52,53] across census tracts in the DMA. To estimate segregation, a two-level variance components binomial response model for proportions, to the 2010 cohort of residents is outlined as:

(1) $$y_{j}\sim \mathrm{Binomial}\left(n_{j},\pi_{j}\right)$$

(2) $$\mathrm{logit}\left(\pi_{j}\right)=\beta_{0}+u_{j}$$

(3) $$u_{j}\sim N\left(0,\sigma_{u}^{2}\right)$$

where $y_{j}$ is the observed proportion of blacks in census tract $j,\;n_{j}$ is the total number of residents in that census tract, and $\pi_{j}$ is the unknown underlying proportion of black residents. The underlying proportion is related to the linear predictor $\beta_{0}+u_{j}$ through a logit link function. Taking the antilogit of $\beta_{0}$ calculated as (exp $\beta_{0}$/(1 + (exp $\beta_{0}$ ))) gives the proportion of black residents in a median census tract. To obtain the proportion of black residents in a mean census tract, simulations were conducted using Markov Chain Monte Carlo (MCMC) estimation with a burn-in of 500 iterations and 250,000 monitoring chain length. The $u_{j}$ are random effects that vary across census tracts. The random effects are considered normally distributed with mean zero and variance $\sigma_{u}^{2}$. The estimate of the variance is the unknown variation in the proportion of blacks across census tracts; and is therefore, the measure of segregation. Larger variances represent greater degrees of black segregation. If there is no segregation, the $u_{j}$ and thus, the $\sigma_{u}^{2}$ are zero. In this study the antilogit of $\pi_{j}$ = (*β_0_ + u_j_*) was calculated and mapped to visualize the spatial patterns of segregation at the census tract level across the DMA.

There were two advantages to using a multilevel model in this study. The first was the ability to control for city-level variation in black residents, thereby reducing the potential for cross-level confounding in the racial distributions [52]. Previous research has shown that segregation measured at the local level may result in an overestimation, if city level segregation is ignored [54]. The second advantage to using multilevel modeling was the ability to estimate segregation independent of poverty. It was hypothesized that segregation would decrease in those areas, where segregation was largely explained by poverty, i.e., those areas of concentrated poverty [55]. A three-level multilevel model was therefore, used to estimate census tract level segregation as described above, controlling for the variation in percent black at the city level and percent poverty at the census tract level (equation not shown). Poverty was calculated using the ratio of family income to poverty level—adding the number of residents in the three lowest groups reflecting severe poverty (ratios < 0.50), poverty (ratios 0.50–0.99) and near poverty (ratios 1.0–1.24) divided by the total population in the census tract. The data on race and poverty were obtained from the U.S. Bureau of the Census American Community Survey 2009–2013 [56]. The estimation of segregation was modeled in MLwiN v. 2.23 (University of Bristol, Senate House, Tyndall Avenue, Bristol, UK) [57]. The segregation measures were mapped using a natural breaks classification scheme in ArcGIS v. 10.3 (Environmental Systems Research Institute (ESRI), Redlands, CA, USA) [58].

### 2.2. Lead Emissions

The Michigan Air Emissions Reporting System (MAERS) requires that annual emission inventory reports be filed by commercial, industrial, or governmental entities for each permitted source (including grandfathered sources) contributing hazardous air pollutants and criteria pollutants (Michigan Rule 336) outlined by the Air Quality Division (AQD) Policy and Procedure number AQD-013 (Michigan Department of Environmental Quality [59]. The monitoring and reporting emission threshold for lead in the State of Michigan is 0.5 tons per year (referenced in Michigan Rule 336.1212 (7)). Allowable emissions are dictated by each facility’s specific individual permit [59]. Stack tests and Continuous Emission Monitoring Systems (CEMS) are used to collect emissions data [60]. The MAERS data (2006–2013) provided the variables for the lead emissions modeling described in the next section.

### 2.3. Lead Emission Modeling 

AERMOD is the U.S. EPA’s advanced plume modeling program with design criteria for regulatory applications. It uses a Gaussian approach to assume that the simulated concentration values follow a bell-shaped distribution where the concentration of the modeled pollutant is highest at the center of the point source’s plume and decreases exponentially approaching zero at the plume edge [61]. AERMOD incorporates air dispersion based on planetary boundary layer turbulence structure and scaling concepts, including treatment of both surface and elevated sources, and both simple and complex terrain. There are two input data preprocessors that are regulatory components of the AERMOD modeling system, AERMET and AERMAP. AERMET is a meteorological data preprocessor that incorporates air dispersion based on planetary boundary layer turbulence structure and scaling concepts using the AERMINUTE and AERSURFACE programs and data from the National Weather Service (NWS). AERMINUTE calculated hourly average wind speeds and directions.

Meteorological data and processing determines three surface characteristics: surface roughness $\left\{z_{0}\right\}$, albedo {r}, and the Bowen ratio $\left\{B_{0}\right\}$. Surface roughness refers to the height of obstacles to the wind flow and its length is important in determining the magnitude of mechanical turbulence and stability of the boundary layer. Albedo is the fraction of total incident solar radiation reflected by the surface without absorption. The daytime Bowen ratio is an indicator of surface moisture (ratio of sensible heat flux to latent heat flux). The albedo and Bowen ratio are used to determine how much incoming radiation is converted to sensible heat flux. In this study, adjusted meteorological data corresponded to the year of emissions; i.e., 2006 meteorological data was applied to the 2006 lead emissions data. In 2015, the U.S. EPA proposed imputing meteorological data that has adjusted surface friction velocity and a low wind option to address issues with AERMOD overprediction under stable, low wind speed conditions [62]. Particle deposition parameters were set at 0.75 for fine mass fraction and 0.5 for the representative mass-mean aerodynamic particle diameter in microns (U.S. EPA’s default values for lead). Surface characteristics parameters range from 0.16 to 0.47 for albedo, 0.49 to 0.96 for the Bowen ratio, and 0.018 to 0.090 for surface roughness length (km). The Detroit Metropolitan Airport (station ID 94847) was utilized for surface weather variables and the National Weather Surface White Lake facility (station ID 4830) for upper air variables.

The second preprocessor is AERMAP used to characterize terrain and generate receptor grids for AERMOD using the United States Geological Service (USGS) Digital Elevation Data [63]. Surface characteristics are also determined using source of elevation, land use, and land cover. In this study, urban versus rural modeling specification were selected. Surface roughness length is related to the height of obstacles to the wind flow and is, in principle, the height at which the mean horizontal wind speed is zero based on a logarithmic profile. When combined with wind speed, the effects are best modeled for stable atmospheric conditions and meteorological monitoring sites are typically open (low roughness) exposures to accommodate siting criteria [63]. The data used to determine the elevation (hills and terrain) and land cover were derived from the USGS’s 30-m National Elevation Dataset (NED).

The domain of a 500-m by 500-m resolution was projected into Universal Transmercator Projection (UTM) Zone 17 coordinate system. The plot file for the aggregate study period of 8 years (17,128 total hours) consisted of 21,136 grid receptors. Predicted airborne and depositional lead concentrations are influenced by surface roughness parameter differences between the facility emission and the meteorological measurement site and will depend on the nature of the application (i.e., the release height, plume buoyancy, terrain influences, downwash considerations, design metric, etc.). Facility-identifying characteristics input into the modeling are displayed in Table 1.

In this study, emission quantities of greater than or equal to 0.1 pound (0.05 kg) per stack per year of atmospheric lead were modeled using AERMOD. Smaller quantities by certain facilities in certain years were initially input but found to be undetectable and therefore, not added in subsequent models. Because fugitive source emission heights were not provided, the authors used a standard industrial facility height of 10 m (or 32.81 feet).

AERMOD’s first output for each receptor included the annual estimated airborne lead presented in μg/m^3^ and the total depositional lead reported in g/m^2^. AERMOD’s second output for each receptor included the monthly maximum levels for airborne lead obtained by dividing the annual pounds of lead emission by 8760 h (in a year) and then incorporating the hourly meteorology to derive maximum monthly and annual lead averages (direct hourly, monthly, and annual outputs are not automated in AERMOD for lead in particular).

In this study, both airborne (wet and dry) and depositional concentrations were mapped individually for each year of this study and compared temporally and across counties in the DMA using bar and line graphs. Some of the key assumptions applied to the dry deposition model included flux rate, concentration of lead, deposition velocity, height of stack, and surface roughness. The wet deposition model included the flux of the particulate, column average concentration of particles in the air, a washout coefficient, and precipitation rate from the meteorological profile [64]. Both the wet and dry flux were calculated on an hourly basis and summed to obtain total flux.

The average residence time of lead in the atmosphere is 10 days [13]; however, lead is extremely persistent in water and soil, thus the accumulation of annual total lead deposition was also calculated and reported over the 8-year study period. The areas of highest airborne and depositional lead were mapped and related to the spatial patterns of racial residential segregation and poverty at the census tract-neighborhood level in the DMA.

The estimated lead emissions gridded-output values from AERMOD were input into ArcGIS v. 10.3 for each of the years of study [58]. The UTM coordinates were converted to a geographic coordinate system (Latitude and Longitude) to more easily overlay onto the neighborhood-characteristics data layers.

### 2.4. Childhood IQ Impacts

Calculated childhood IQ losses and the map classification scheme for airborne lead were established using the EPA’s Air-Related IQ Loss Assessments [65,66]. Airborne lead emissions are inhaled and then absorbed by the body resulting in reduced neurocognitive function in terms of IQ (Intelligence Quotient). The U.S. EPA used a biokinetic concentration response function to inform updated National Ambient Air Quality Standards (NAAQS) for point sources of lead in the latest regulatory review, using AERMOD to dispersion model these point sources [66]. Air-related population mean IQ loss for children exposed at varying levels of the lead standard (from 0.02 to 0.50 μg/m^3^) were estimated using air-to-blood ratios of 1:3, 1:5, and 1:7. These values were established from pooled analysis of many epidemiological studies that compared populations living in different locations with varying ambient air lead levels. The form of the function used by the EPA also recognizes that a steeper slope exists at lower BLLs, in a loglinear way, with lower exposures yielding greater IQ loss [65]. As such, higher versus lower blood lead options exist for each air-to-blood slope ratio because the pooled analysis revealed that lower BLLs have even steeper slopes related to IQ loss than those observed in studies involving higher BLLs [65,66]. The concentration response (C-R) function calculation is derived from multiple studies that examined the relationship between IQ loss and BLLs in children at less than 10 μg/dL and is as follows [66], (Table 1):

I = LBS(4)

where I is the mean air related IQ loss for children exposed concurrently at the time of level of the standard, L is the level of the lead standard (μg/m^3^), B is the air-to-blood ratio in units of μg/dL BLL per μg/m^3^ air concentration, and S equals the slope factor for the C-R function as points of IQ loss per μg/dL BLL. This function is given emphasis in the risk assessments considered by the EPA for children with BLL concentrations below 10 μg/dL [65,66]. For this analysis, the modeled annual airborne lead emissions were used. Air-to-blood ratios (B) and slope values (S) were referenced from Table 1 of U.S. EPA’s *Air-Related IQ Loss Evidence-Based Framework and Estimates* document [66] (see Table 2 below). Children living in neighborhoods of the DMA from 2006 to 2013 exhibited geometric mean BLLs of 10 μg/dL or less [11,12].

The map classification scheme for depositional lead was developed using the same sources [65,66]. Contemporary rates of total lead loadings to terrestrial ecosystems are estimated to be approximately 1 to 2 μg/m^2^ per year. Although these breakpoints are not human health thresholds, the depositional lead loadings in/on soils may be subsequently contacted and ingested by children and taken up by plants in urban garden settings. Additionally, resuspension of soil-bound lead particles is transported back into the air and/or incorporated in house/school dust. Following the modeling and mapping of estimated lead emissions—the three highest years of total airborne lead (2007, 2008, and 2010) and deposited lead for the 8-year period were mapped.

### 2.5. Segregation and Lead Concentrations 

Since 2007 had the highest total airborne emission levels, these data were spatially joined to the census tract segregation GIS layer. Bivariate plots were created to visualize the spatial autocorrelation between airborne lead and increasing levels of segregation determined by the natural breaks classification scheme. These plots were created in the GeoDa freeware software (The University of Chicago, Center for Spatial Data Science, Computation Institute, 5735 S Ellis Ave, Room 230, Chicago, IL, USA available at https://spatial.uchicago.edu/software).

## 3. Results

Figure 1 is a reference map that shows the AERMOD domain and locations of lead emissions facilities. Of the 78 stationary facilities emitting lead, 41 (52%) were in Wayne County, 19 (24%) were in Oakland County and 17 (22%) were in Macomb County. There were 12 facilities in the city of Detroit and 15 facilities on the western and southwestern periphery of Detroit representing 66% of facilities in Wayne County. In Oakland County the facilities were concentrated around the cities of Pontiac, Novi, Wixom, Auburn Hills, and Rochester Hills. In Macomb County the lead emitting facilities followed the north-south State Highway M-53. These facilities in general extended across the study domain. Of these 78 facilities, 48 facilities (61.5%) had more than one emissions stack or fugitive source (an industrial process not captured or vented through a stack). Unlike the federal EPA Toxic Release Inventory data requirements for emissions reporting, the MAERS state data must enter a quality assurance/quality control program and is based on the lead emission activity and devices of each facility’s point sources (usually stacks) [60].

### 3.1. Study Area and Population Vulnerability

**Figure 2:**
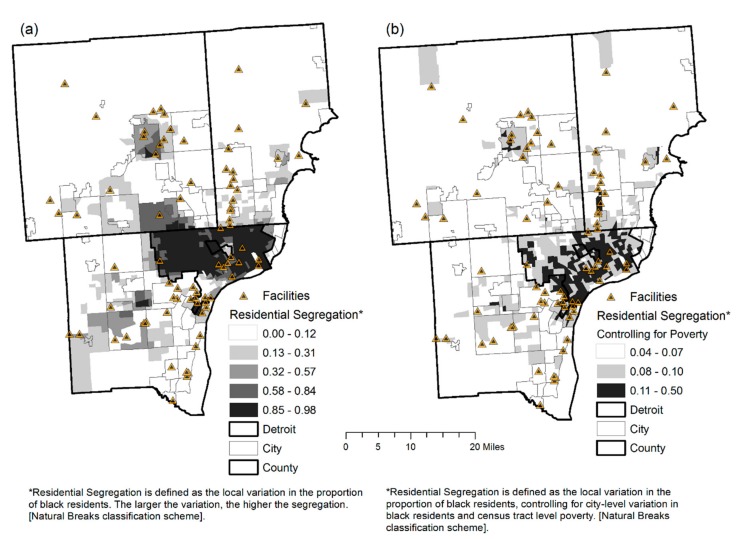
Reference maps—racial residential segregation across census tracts in the Detroit Metropolitan Area, 2010. (**a**) residential segregation is defined as the local variation in the proportion of black residents. The larger the variation, the higher the segregation. (**b**) residential segregation is defined as the local variation in the proportion of black residents, controlling for city-level variation in black residents and census tract level poverty.

Figure 2a,b show the spatial patterns of racial residential segregation at the census tract-neighborhood level in the DMA. Importantly, when city-level variation in black residents and poverty were controlled for in the segregation model, segregation across the study area was greatly reduced, particularly in neighborhoods extending into northwest Detroit and Southfield. Segregation remained high across Southwest Detroit.

### 3.2. Lead emissions-Counties of Changing and Highest Concentrations

**Figure 3:**
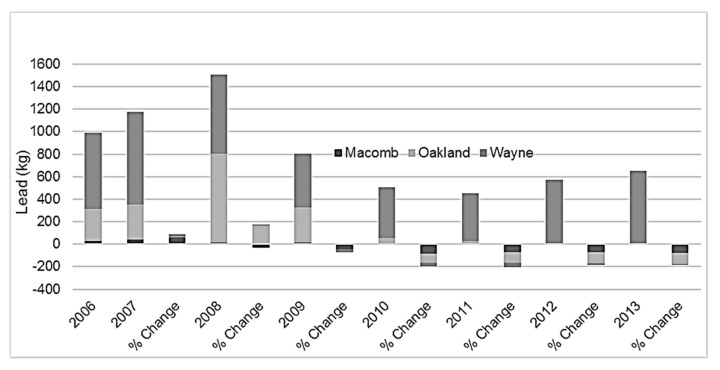
Total reported lead emissions and percent change by county using 2006 as the baseline, Detroit Metropolitan Area, 2006–2013.

The highest lead emissions by industry during the study period are displayed on Table 3. As demonstrated in Figure 3, percent change from 2006 annual lead emissions compared to each subsequent year in Macomb and Oakland Counties shows a decrease over time throughout the study period. Wayne County originally experienced a decline in lead emissions but has since endured a resurgence of consistently higher emissions throughout the later years of the study period.

### 3.3. Lead Emission Modeling 

**Figure 4:**
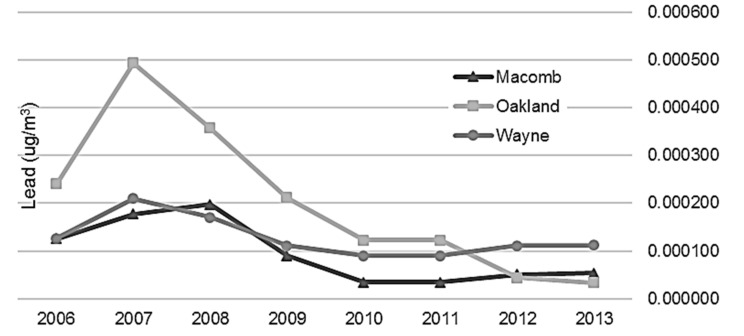
Average airborne lead concentration by county and year, Detroit Metropolitan Area, 2006–2013.

**Figure 5:**
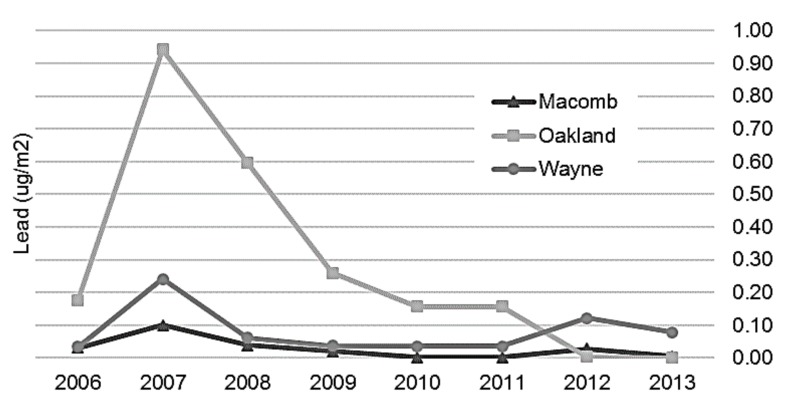
Average depositional lead concentration by county and year, Detroit Metropolitan Area, 2006–2013.

**Figure 6:**
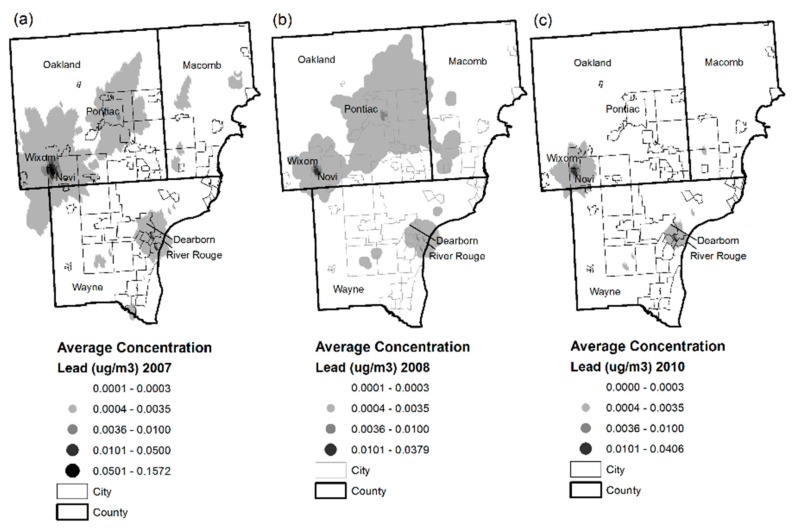
Three highest airborne lead concentration years (2007, 2008, 2010) over the study period in the Detroit Metropolitan Area. (**a**) average concentration lead in 2007; (**b**) average concentration lead in 2008; (**c**) average concentration lead in 2010.

Modeling of point source emissions (Figure 4 and Figure 5) revealed that during the beginning of the study period from 2006 to 2009, total airborne and depositional lead concentrations were highest in Oakland and Wayne Counties, especially Novi to Wixom, Pontiac, and southwest Detroit between Dearborn and River Rouge (see Figure 6 for reference). Beginning in 2010 and continuing through 2013, airborne and depositional lead concentrations shifted away from Oakland and Macomb Counties to Wayne County.

Figure 6a–c show the spatial patterns of the three highest years of resultant modeled airborne lead values over the study period. As described above, airborne lead concentrations shifted to Wayne County neighborhoods described above.

**Figure 7:**
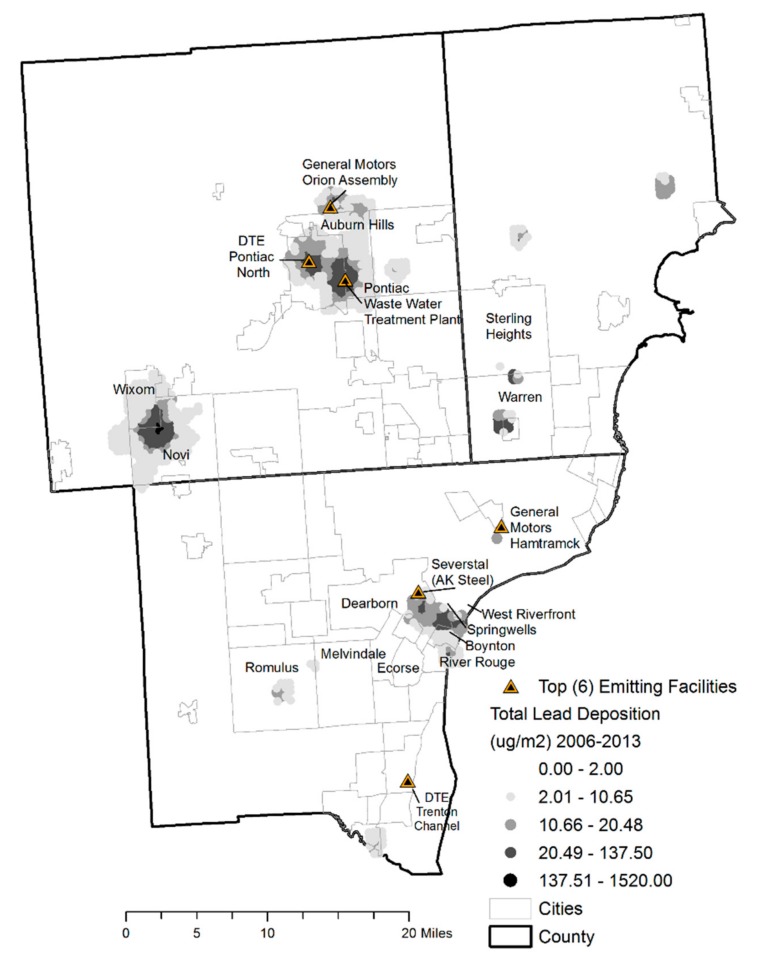
Total lead deposition, AERMOD domain, Detroit Metropolitan Area, 2006–2013.

**Figure 8:**
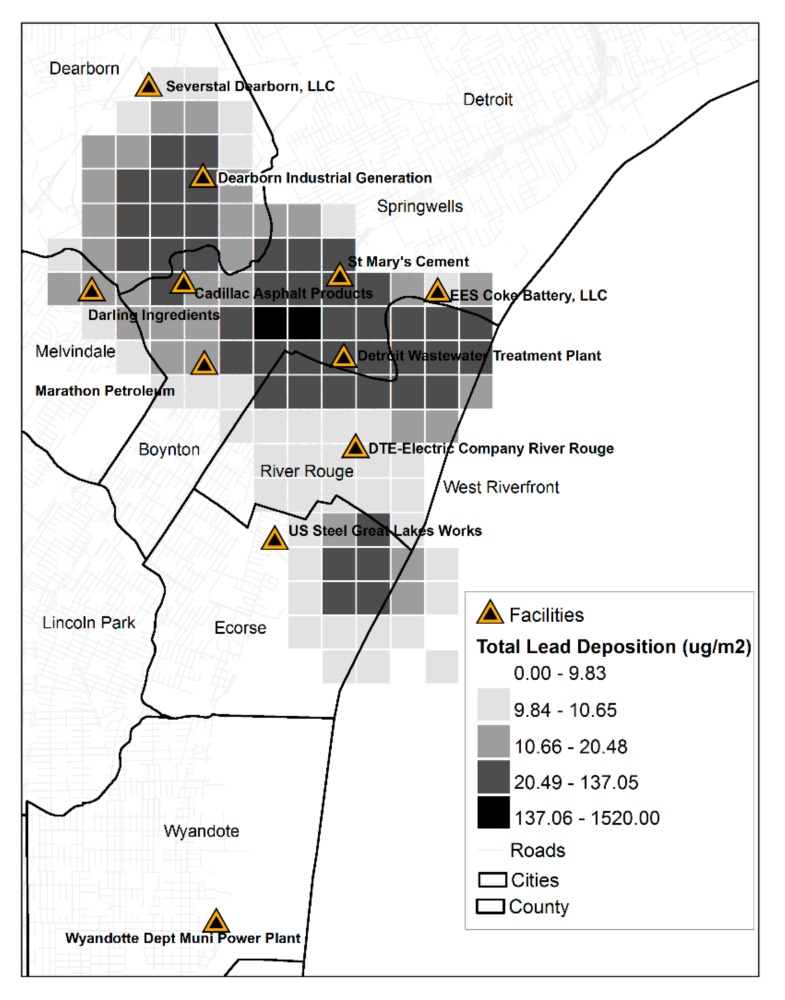
Total lead deposition in Southwest Detroit and surrounding suburbs, 2006–2013.

The total depositional lead values over the 8-year study period were summed and mapped (Figure 7 and Figure 8). Those areas with the highest accumulation of lead were in the mostly black segregated communities of Boynton, Springwells and West Riverfront neighborhoods in Southwest Detroit (~2043.1 μg/m^2^) and surrounding suburbs of Detroit—River Rouge, Ecorse, and Melvindale and in the largely white-Arab population of Dearborn (~1860 μg/m^2^). Similar findings were observed in the cities of Novi-Wixom and Pontiac and Auburn Hills where cumulative lead deposition ranged from 1890 to 10,410 μg/m^2^, in the more black segregated neighborhoods across these four cities. There was cumulative lead deposition of approximately 330 μg/m^2^ in other black segregated cities along Highway M-35 including Sterling Heights, Warren, and Romulus (~230 μg/m^2^) that contains three facilities, including the Detroit Metropolitan Airport. Considering all reported emissions and resultant modeled lead concentrations, from 2006 to 2009, the Tony Angelo Cement Construction Company contributed to the airborne and depositional plumes from Novi to Warren while the Pontiac Wastewater Treatment Plant and Detroit Edison Pontiac North coal burning facility were the contributors in Pontiac. The shifting of greater emissions to Wayne County was the result of large and/or increased lead emissions from facilities including General Motors Hamtramck, the Southwest Detroit Wastewater Treatment Plant sewage burning facility, Greater Detroit Resource Recovery municipal waste burning facility, Detroit Edison River Rouge/Ecorse coal burning facility, Trenton Channel coal burning facility, EES Coke Battery facility of River Rouge/Ecorse, Romulus Ajax Materials Corp., and Severstal (AK Steel) of Dearborn.

As a result black segregated areas of these recipient neighborhoods were impacted (see Figure 6 for reference). By the end of 2013, 9 of the 10 highest lead emitting facilities were in Wayne County in these areas.

### 3.4. Childhood IQ Impacts 

Beginning in 2007, lead air concentrations reached levels evidenced to effect neurocognitive function loss associated with alternative levels of the air quality standard for lead. The highest lead air concentration was 0.16 μg/m^3^ found in Novi bordering Wixom—translating to an IQ loss of 1.0–1.8, 1.7–2.9, or 2.7–3.0 using the lower bound concentration response function and air-to-blood slope ratios of 1:3, 1:5, and 1:7 in children living under this plume [66] (Table 2). The NAAQS for lead is 0.15 μg/m^3^, in terms of a 3-month average concentration and would result in an estimated mean childhood IQ loss of 1.8 points [66]. But other epidemiological research finds that children of socioeconomic disadvantage display greater susceptibility to environmental toxins and in particular, Chari et al. find that such vulnerable populations would experience greater declines at the 0.15 μg/m^3^ level, equal to approximately a 2.5 loss of IQ points [67]). In 2008 and 2010, airborne lead concentrations in the Novi and Wixom area reached 0.04 μg/m^3^ both, translating to a childhood IQ loss of 0.2–0.3, 0.3–0.4, or 0.4–0.6 (air-to-blood slope ratios of 1:3, 1:5, and 1:7) [66] (Table 2). By 2011, airborne lead concentrations in these areas became non-detectable and high lead emissions of Tony Angelo’s Cement Construction Company ceased.

On the other hand, throughout the entire study period, Dearborn and South/Southwest Detroit consistently reached airborne lead concentrations of 0.01 μg/m^3^. In combination with the proven higher BLLs in these areas [11,12], IQ deficits are predicted. The corridor between General Motors in Hamtramck extending to Southwest Detroit and its suburbs experience intermittent elevated airborne lead emissions but have high lead deposition values exposing residents and their children who reside in this area to lead laden dirt and dust from roads and yards as it enters the home and schools upon resuspension.

### 3.5. Segregation and Lead Concentrations 

**Figure 9:**
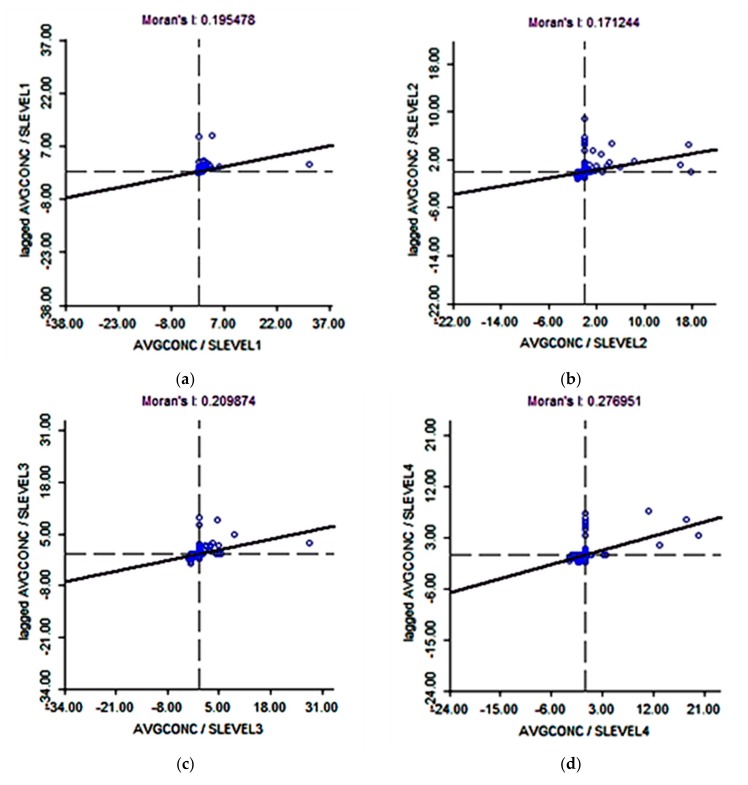

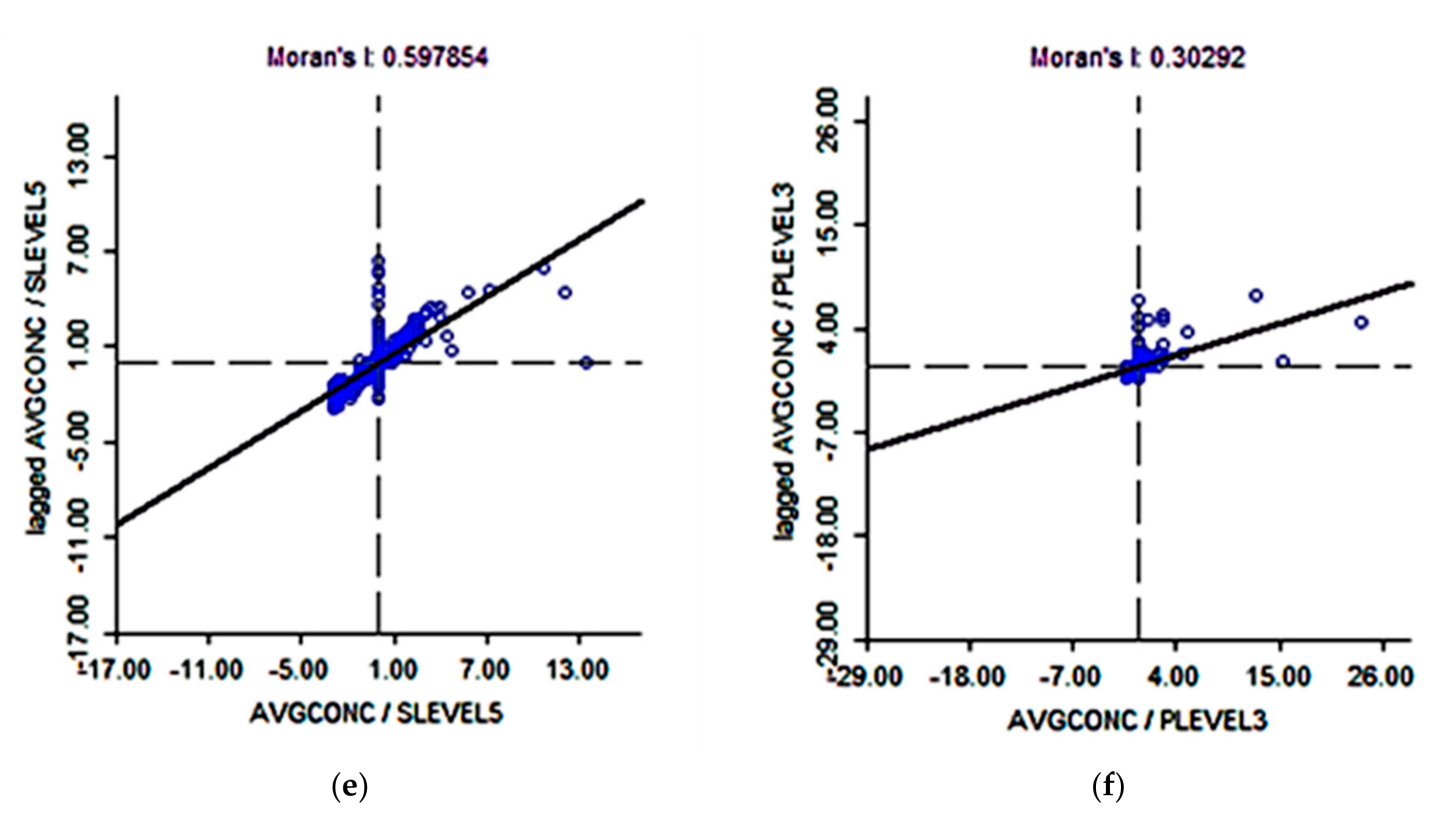
(**a**–**e**) Bivariate spatial autocorrelation of airborne lead concentrations (AVGCONC) (2007) and five levels (Natural Breaks classification scheme) of segregation (SLEVEL1-5). (**f**) Controlling for city-level racial differences and poverty (PLEVEL3).

To support the visual findings above, the association between airborne lead concentrations and black segregation were statistically analyzed. Bivariate spatial autocorrelation plots of airborne lead concentrations and five levels of segregation (Figure 9a–f) show that in low segregated neighborhoods, the air concentrations of lead were also low and with increasing segregation, airborne lead concentrations rise. For example, at the lowest level of segregation the Moran’s I = 0.1954 (Figure 9a) compared to the highest level of segregation Moran’s I = 0.5978 (Figure 9e). Figure 9f shows the bivariate spatial autocorrelation for airborne lead and the highest level of segregation, controlling for city level racial differences and neighborhood level poverty Moran’s I = 0.3029. These findings suggest that segregation is a more important determinant of airborne lead than poverty.

## 4. Discussion

The DMA is indicative of the environmental injustice suffered by minority segregated and impoverished communities across the United States. This study also found that airborne lead and lead deposition mostly impact the vulnerable in highly segregated black neighborhoods of the DMA, even after controlling for poverty. As segregation increased so did airborne lead concentrations; showing that segregation was a greater determinant of high airborne lead emission levels than poverty. Furthermore, evidence supports that lead emitting facilities and lead emissions moved from black segregated communities of Pontiac in Oakland County to poorer and more black segregated communities of Wayne County, especially Dearborn, River Rouge, Ecorse and Southwest Detroit.

Auto manufacturing and supportive mineral industries, infrastructure, and power supplies of Detroit began in earnest in the early 20th century and continued growing until 1953. These industries attracted workers from other parts of the state, nation and even immigrants from Europe and the Middle East [68]. Black and immigrant neighborhoods grew in close proximity to the manufacturing plants in Detroit and subsequently Dearborn. However, as the auto industry began to disperse or decentralize to the suburbs (from 1953 to 2005), the City of Detroit’s urban infrastructure decayed. Residential development of the DMA ensured that black neighborhoods remained in the declining (in terms of investment, jobs, and infrastructure) urban core, while whites benefited from government subsidized housing in the suburbs [69]. As a result, black residential segregation became the most extreme of any metropolitan area in the U.S. [69] (see Figure 2). With extreme racial residential segregation came extreme class segregation [69]. Poor black neighborhoods were left in close proximity of abandoned industrial pollutants [9,10] and the most undesirable lead emitting facilities [11,12].

Also indicative of environmental injustice is the practice of burning municipal waste (i.e., Greater Detroit Resource Recovery facility) and sewage sludge (i.e., Detroit’s Wastewater Treatment Plant) as an energy recovery cost savings measure. White affluent communities possessed with oppositional power are rarely located near such practices and their resultant emissions. Children residing in the black segregated neighborhoods of Novi and Wixom, up until 2011, were exposed to high lead airborne concentrations predicting IQ loss. Those residing in the most black segregated communities of southwest Detroit continue to experience constant airborne lead and depositional levels predicting IQ loss; an untoward risk that children residing in more white segregated and affluent neighborhoods do not experience. Finally, the lack of enforcement of existing environmental laws and cleanup standards is another characteristic of environmental injustice. This is demonstrated by the lack of regulation by the MDEQ of the DMA’s steel plant, refinery, and smelter, all located in highly segregated and impoverished black and Arab communities.

The predicted IQ loss of children living in the DMA applies only to the initial effects of airborne lead inhaled and does not address the effects of ingestion of lead deposited in dust/soil or taken up in drinking water or other environmental lead bioaccumulations past or prior. Further, the IQ values calculated are likely underestimated as the model under predicts lead exposure, as current lead environments are not incorporated. Further, research is revealing even greater IQ impacts of children living with socioeconomic disadvantage.

This research built upon the environmental injustice and neighborhood effects literature, particularly of the Detroit Metropolitan Area. Mohai and Bryant [5] found black and impoverished residents to be in closer proximity to polluting industries or in Downey’s research, toxic releases [8] and in Smith’s research [10] Superfund site locations. Keeler [6] and Wu and Batterman [7] found closer proximity of black and respectively non-white poor residents to traffic particulate matter. Lee and Mohai [9] found Brownfield sites located in poor minority neighborhoods. In this study, highly segregated minority residents were exposed to greater industrial lead emissions, even after controlling for poverty. Pizzol et al. [70] too calculated the external costs in terms of childhood IQ loss related to airborne lead emissions also using a Gaussian plume dispersion model. This study however, was the first to examine airborne and depositional lead using a similar and rigorous Gaussian plume dispersion model but also related the resultant concentrations to neighborhood segregation and poverty. Furthermore, it was the first to apply the airborne emissions by using a biokinetic model for children to determine IQ loss as related to these neighborhoods.

### Limitations

The concentration response function of airborne lead emission to IQ loss was applied only to the initial health effects of inhaling leaded air directly from the source. Other sources such as ingested and inhaled leaded dust and soils were not estimated or added to this exposure risk but certainly effect neurocognitive function in children exponentially. For example, the Master Metal battery smelter (NL Industries, Dallas, TX, USA) that emitted illegal amounts of lead dust for two decades on Detroit’s segregated poor and minority east side would not be reflected in our model as it ceased operations in 1984. Deposition levels are underestimated in this area given the lack of remediation and the stability of lead dust.

The MAERS emissions data are not an exhaustive source of all lead air pollution. Industries are required to report information to MAERS only if they are a listed industrial activity by MDEQ standards. Other small sources of lead emissions may exist in the DMA but these facilities are not obligated to report [59]. Second, the modeled values are estimates for each facility’s lead emitting activity regulated by the MDEQ. AERMOD incorporates annual and seasonal emission rates of permitted facilities and applies these rates over a 12-month period to characterize monthly and annual lead impact concentrations. The EPA has not established confidence intervals or error values associated with these AERMOD parameters or output values. However, the model has been tested extensively prior to adoption as a regulatory tool.

## 5. Conclusions

This study found that from 2006 through 2013, elevated air lead emission concentrations and deposition shifted from black segregated communities of Pontiac and surrounding areas in Oakland County to even greater levels of black segregation in Southwest Detroit, River Rouge, and Ecorse and Arab neighborhoods of Dearborn in Wayne County. A trend of weakening permit conditions allowing for increased lead emissions combined with a lack of enforcement power, subject children of these communities to greater exposure and IQ loss, demonstrating environmental injustices in the DMA. Across the DMA annual lead deposition values were substantially higher than recommended for terrestrial impacts, further exposing these communities to resuspension of lead particles and additional IQ loss. These findings should inform current and future regulators as to the injustice and largely preventable nature of lead exposure to children in the Detroit Metropolitan Area. Future research will include these findings in relation to racial disparities in childhood blood lead levels and build on previous health disparity research in the DMA [71,72] to include the contribution of maternal lead exposures on adverse birth outcomes.

## Figures and Tables

**Table 1:** AERMOD facility identifying information for the largest emitter of lead **^1^** over the study period in the Detroit Metropolitan Area, 2006–2013.

Year	Emission Unit Stack Identifier	Process Identifier	USEPA Source Classification Code	Reported Annual Emissions Lead Pounds (kg) ^2^	Release Point Stack Name ID	Release Point Orientation	Stack Height (ft)	Stack Diameter (ft)	Exit Gas Temp. (°F)	Exit Gas Flow Rate (ft^3^/min)	Exit Gas Velocity (ft/s)
2006	RG0096	PR0001	10200602	0.05 (0.02)	SV0002	Vertical	250	10	380	5421.33	69
	RG0096	PR0002	10200204	804.27 (364.81)	FUG001	Fugitive	32.81				
2007	RG0096	PR0001	10200602	0.05 (0.02)	SV0002	Vertical	250	10	380	5421.33	69
	RG0096	PR0002	10200204	768.09 (348.40)	FUG001	Fugitive	32.81				
2008	RG0096	PR0001	10200602	0.01 (0.004)	SV0002	Vertical	250	10	380	5421.33	69
	RG0096	PR0002	10200204	726.12 (329.36)	FUG001	Fugitive	32.81				
2009	RG0096	RGPOWERHOUSE	10200204	436.51 (198.00)	SV043	Vertical	250	120	380	325,280.00	69.03
	RG0096	RGPOWERHOUSE	10200602	0.04 (0.02)	Non Listed	Non Listed	NA	NA	NA	NA	NA
2010	RG0096	RGPOWERHOUSE	10200204	442.71 (200.81)	SV043	Vertical	250	120	380	325,280.00	69.03
	RG0096	RGPOWERHOUSE	10200602	0.04 (0.02)	Non Listed	Non Listed	NA	NA	NA	NA	NA
2011	RG0096	RGPOWERHOUSE	10200204	435.37 (197.48)	SV043	Vertical	250	120	380	325,280.00	69.03
	RG0096	RGPOWERHOUSE	10200602	0.04 (0.02)	Non Listed	Non Listed	NA	NA	NA	NA	NA
	RG0126	RGTEMPBOILERS	10200602	0.002 (0.001)							
2012	RG0096	RGPOWERHOUSE	10200204	356.74 (161.81)	SV043	Vertical	250	120	380	325,280.00	69.03
	RG0096	RGPOWERHOUSE	10200602	0.04 (0.02)	Non Listed	Non Listed	NA	NA	NA	NA	NA
2013	RG0096	RGPOWERHOUSE	10200204	439.40 (199.31)	SV043	Vertical	250	120	380	325,280.00	69.03
	RG0096	RGPOWERHOUSE	10200602	0.01 (0.004)	Non Listed	Non Listed	NA	NA	NA	NA	NA

**^1^** General Motors Hamtramck; **^2^** Spring, Summer, Fall, Winter lead emissions varied from 0 to 100% per stack.

**Table 2:** Estimates of air-related population mean IQ loss for children exposed at the level of the standard. **^1^** These estimates were derived using only the nonlinear C-R function from the risk assessment which, given its nonlinearity, is considered to better assess risk across the range that includes extending into these higher standard levels (and the associated higher blood Pb levels).

Potential Level for Standard (g/m^3^)	Air-Related Population Mean IQ Loss (Points) for Children Exposed at Level of the Standard					
	Air-to-Blood Ratio of 1:3		Air-to-Blood Ratio of 1:5		Air-to-Blood Ratio of 1	
	1st Group of C-R Functions (From Lower Blood Pb Analyses)	2nd Group of Functions (from Higher Blood Pb Analyses)	1st Group of C-R Functions (From Lower Blood Pb Analyses)	2nd Group of C-R Functions (From Higher Blood Pb Analyses)	1st Group of Functions (From Lower Blood Pb Analyses)	2nd Group of Functions (From Higher Blood Pb Analyses)
0.50	2.9–3.1	1.4	4.1–4.3	2.3	5.0–5.3	3.2
0.40	2.4–2.6	1.1	3.5–3.8	1.8	4.4–4.6	2.5
0.30	1.5–2.6	0.8	2.9–3.1	1.4	3.6–3.9	1.9
0.20	1.0–1 8	0.5	1.7–2.9	0.9	2.7–3.0	1.3
0.10	0.5–09	0.3	0.9–1.5	0.5	1.2–2.1	0.6
0.05	0.3–0.4	0.14	0.4–0.7	0.2	0.6–1.0	0.3
0.02	0.1–0.2	0.05	0.2–0.3	0.09	0.2–0.4	0.1

**^1^** Table 1 of U.S. EPA’s Air-Related IQ Loss Evidence-Based Framework and Estimates document [66].

**Table 3:** Largest lead emitters in the Detroit Metropolitan Area by county, 2006–2013.

Facility	Lead Emissions (kg) ^1^	County
General Motors Hamtramck, Hamtramck	2000.11	Wayne
Severstal Dearborn, L.L.C., Dearborn (AK Steel)	889.60	Wayne
Detroit Edison Co., Pontiac North, L.L.C., Pontiac	741.95	Oakland
Detroit Edison Co., Trenton Channel, Trenton	639.76	Wayne
Pontiac Wastewater Treatment Plant, Pontiac	509.68	Oakland
General Motors Corp., Orion Assembly, Lake Orion	364.47	Oakland
St. Mary’s Cement, Detroit	239.40	Wayne
Tony Angelo Cement Construction Co., Novi	217.69	Oakland
U.S. Steel Great Lakes Works, Ecorse	215.43	Wayne
Greater Detroit Resource Recovery, Detroit	188.91	Wayne
EES Coke Battery, L.L.C., River Rouge	179.89	Wayne
Detroit Edison River Rouge, River Rouge	132.46	Wayne
Detroit Wastewater Treatment Plant, Detroit	103.36	Wayne
Cadillac Asphalt Shelby Plant, Shelby	65.93	Macomb
ACE Asphalt & Paving Co. Inc., Davisburg	41.79	Oakland
Cadillac Asphalt, L.L.C., Clarkston	36.48	Oakland
Warren Waste Water Treatment Plant, Warren	35.54	Macomb
Ajax Materials Corp., Romulus	9.87	Wayne
Ajax Materials Corp., Warren	8.82	Macomb
Ajax Materials Corp., Rochester	8.47	Oakland
Ajax Materials Corp., Auburn Hills	8.39	Oakland
Ajax Materials Corp., New Haven	8.26	Macomb
Marathon Petroleum Company, LP, Detroit	7.62	Wayne
Ajax Materials Corp., Rockwood	2.71	Wayne
Ajax Metal Processing, Detroit	0.28	Wayne

**^1^** Total lead emissions for facilities by county from 2006 to 2013. Total = 6656.88 kg; Source: Michigan Air Emissions Reporting System (MAERS).
